# Dutch guideline for preventing nosocomial transmission of highly-resistant micro-organisms (HRMO) in long-term care facilities (LTCFs)

**DOI:** 10.1186/s13756-019-0586-3

**Published:** 2019-08-27

**Authors:** Andrea Eikelenboom-Boskamp, Jobje Haaijman, Maria Bos, Katja Saris, Else Poot, Andreas Voss, On behalf of the expert group ‘Long-term care facilities’ of the former Dutch Working Party on infection control, E. Stobberingh, E. Gorissen-Douven, P. Molenaar, R. Hoentjen, P. Tolsma, I. Verzijl

**Affiliations:** 1 Canisius-Wilhelmina Hospital, Department of Medical Microbiology and Infectious Diseases, Nijmegen, The Netherlands; 2 River Region Elderly Care Center (SZR), Tiel, The Netherlands; 3 Avans University of Applied Sciences, Breda, The Netherlands; 4 Verenso Dutch Association of Elderly Care Physicians and Social Geriatricians, Utrecht, The Netherlands; 5 Radboud University Medical Centre, Department of Medical Microbiology, Nijmegen, The Netherlands

**Keywords:** Highly-resistant micro-organisms, HRMO, Antibiotic resistance, Antimicrobial resistance, Long-term care facilities, LTCF, Infection control precautions, Guidelines

## Abstract

In 2012, the Dutch Working Party for Infection Control (WIP) issued the first Guideline for prevention of transmission of highly-resistant micro-organisms (HRMO) in Hospitals. The next step was to focus on long-term care facilities (LTCFs) both for nursing homes as for small-scale living facilities with nursing home care. These facilities providing care for residents with functional disabilities, chronical illnesses and cognitive disorders, such as dementia. The objective was to adapt the Guideline for prevention of transmission of HRMO in hospitals to LTCFs with a strong accent on living conditions and social interactions.

Residents of LTCFs may be carriers of HRMO over a long period of time and most of the residents of the LTCF stay for extended periods of time. To respect individual living circumstances and to prevent unnecessary limitations in the social life of the residents due to the use of isolation measures, the WIP has chosen to describe infection control precautions per individual micro-organism instead of a ‘one size fits all’ method. The term “isolation” was therefore replaced by the term “additional” precautions. This guideline describes the screening policies for residents in LTCFs, definition and detection of HRMO carriage, standard and additional infection control precautions for HRMO positive residents, documentation and communication of HRMO carriage and discontinuation of additional infection control precautions. It also describes contact tracing of HRMO, environmental control/investigation, surveillance of HRMO and what is important when there is an outbreak.

## Introduction

Antimicrobial resistance (AMR) is a worldwide threat to healthcare as common empiric antibiotics may no longer be effective to treat infections, including those that are life-threatening. Consequently, AMR may result in increased morbidity, mortality and cost of healthcare.

The World Health Organization (WHO) issued the Antimicrobial Resistance, Global Report on surveillance in 2014. This report summarizes all information on AMR and speaks of alarming levels of AMR in many parts of the world [1]. In the Netherlands, AMR has, with a few exceptions, stayed on the same level from 2010 to 2015 [2]. Still, to control the increase in AMR, antibiotic should be used wisely and infection control precautions should be installed to prevent transmission of Highly Resistant Micro-Organisms (HRMO).

In 2012, the – by now former - Dutch Working Party for Infection Control (WIP) issued the first Guideline for prevention of transmission of HRMO in Hospitals [3, 4]. This guideline provides definitions for classification of HRMO and recommendations on surveillance, isolation precautions for patients and advice on outbreak management. It is currently implemented in Dutch hospitals. The next step was to focus on long-term care facilities (LTCFs), providing care for residents with functional disabilities, chronical illnesses and cognitive disorders, such as dementia. In the Netherlands, these residents are in the care of an “elderly care physician”, a distinct medical specialization, exclusively working in LTCFs. Within the structure of the WIP, a so-called “Expert group LTCFs” was established, with professionals working (partially) in/for LTCFs. The expert group was tasked with the development of multiple Infection prevention and control guidelines, the first of which was ‘the prevention of transmission of HRMO in LTCFs’.

Based on the Guideline for prevention of transmission of HRMO in hospitals, the ultimate goal was to adjust this guideline [5] to the living circumstances in LTCFs. This concerns all forms of nursing home care within institutions such as nursing homes or small-scale living facilities with the exception of geriatric departments of a hospital. These facilities have a strong emphasis on living conditions and social interactions. As residents of LTCFs may be carriers of HRMO over a long period of time [6] and as most of the residents of the LTCF stay for longer periods of time, infection control precautions may have a negative impact on the quality of life [7]. In order to respect individual living circumstances and to prevent unnecessary limitations in social life, the WIP has chosen to describe infection control precautions per individual micro-organism instead of a ‘one size fits all’ method. In addition, the term “isolation” was replaced by the term “additional precautions”.

This guideline focusses on the control of HRMO and not for control of Methicillin-resistant *Staphylococcus aureus* (MRSA) for which a separate guideline is available in the Netherlands.

## Screening policies for residents in long-term care facilities

When a resident is admitted to a LTCF and has stayed in a healthcare facility outside the Netherlands, HRMO screening must be initiated under the following circumstances:

- When the resident was admitted to a foreign health care facility (outside the Netherlands, the Caribbean islands not included) in the 2 months prior to admission to the LTCF and
- When the duration of admission in a foreign health care facility was longer than 24 h

It is also advised to test a resident for HRMO carriage if the resident is transferred from a ward or small-scale living group within the healthcare facility or another healthcare facility with an ongoing HRMO outbreak within the Netherlands.

### Definition and detection of HRMO carriage

The definition of HRMO is determined by the micro-organism and the specific antibiotic where the micro-organism has shown resistance to. The criteria for HRMO are based on the guideline “Laboratory detection of highly resistant microorganisms (HRMO)” of the Dutch society for Medical Microbiology [8]. This way, the definition of HRMOs are consistent with the established HRMO guideline for hospitals, making adequate information exchange easier [3, 4].

Three main groups of HRMO are distinguished: highly resistant Enterobacteriaceae (Table 1); highly resistant Gram-negative nonfermenters (Table 2), and highly resistant Gram-positive bacteria (Table 3).

**Table 1:** Definition of highly resistant Enterobacteriaceae

Gram-negative rods	ESBL	Carbapenemase	Aminoglycosides	Quinolones
Enterobacteriaceae	A	A	B	B

*ESBL* extended-spectrum beta-lactamase; A: presence of ESBL or Carbapenemase is sufficient to define the microorganism as highly resistant; B: resistance against both antibacterial agents from the two indicated groups is required to define the microorganism as highly resistant

**Table 2:** Definition of highly resistant gram-negative nonfermenters

Gram-negative nonfermenters	Carbapenemase	Aminoglycosides	Quinolones	Ceftazidime	Piperacillin	Co-trimoxazole
*Acinetobacter spp.*	A	B	B^a^	n/a	n/a	n/a
*Stenotrophomonas* *maltophilia*	n/a	n/a	n/a	n/a	n/a	A
*Pseudomonas* *aeruginosa*	C	C	C	C	C	n/a

^a^only Ciprofloxacin and/or Levofloxacin, due to the intrinsic resistance of Acinetobacter species for norfloxacin; A: Carbapenemase or resistance against an antibacterial agent from the indicated group is sufficient to define the microorganism as highly resistant; B: resistance against antibacterial agents from at least two indicated groups is required to define the microorganism as highly resistant; C: resistance against antibacterial agents from at least three of the indicated groups is required to define the microorganism as highly resistant; n/a: not applicable

**Table 3:** Definition of highly resistant gram-positive bacteria^a^

Gram-positive bacteria	Penicillins	Vancomycin
*Streptococcus pneumoniae*	A	A
*Enterococcus faecium*	B	B

^a^: MRSA not included; A: resistance against an antibacterial agent from the indicated group is sufficient to define the micro-organism as highly resistant; B: resistance against both antibacterial agents from the two indicated groups is required to define the microorganism as highly resistant

To detect residents that carry HRMO, specific cultures have to be taken (Table 4).

**Table 4:** Diagnostic screening procedure for residents suspected for HRMO carriage in LTCF

Micro-organism/Indication	*Standard* Cultures^a^	Additional cultures (when indicated)^a,b^
Enterobacteriaceae (ESBL and CPE inclusive)	Rectal swab or stool sample	Wound swab, sputum sample, urine sample
*Acinetobacter species*	Rectal swab or stool sample and sputum sample or oropharyngeal swab^c^	Wound swab, urine sample
*Stenotrophomonas maltophilia*	Rectal swab or stool sample and sputum sample or oropharyngeal swab^c^	Wound swab, urine sample
*Pseudomonas aeruginosa*	Rectal swab, stool sample and sputum sample or oropharyngeal swab^c^	Wound swab, urine sample
*Streptococcus pneumoniae*	Sputum sample or oropharyngeal swab^c^	–
*Enterococcus faecium*	Rectal swabs or stool samples	Wound swabs, sputum samples, urine samples
When resident is transferred from health care facility outside the Netherlands	Rectal swab or stool sample and sputum sample or oropharyngeal swab^c^	Wound swab, sputum sample, urine sample

^a^Single swab/sample from the stated site, excepting for Enterococcus faecium. Standard and additional cultures for Enterococcus faecium: five swabs/samples on five consecutive days

^b^Depending on clinical presentation of the signs and symptoms of resident: ▪ culture of sputum when resident has a persistent cough ▪ culture of wound if present ▪ urine culture when urinary tract catheter is in place

^c^ Preferably sputum sample. If sputum sample cannot be obtained, collect oropharyngeal swab

### Standard and additional infection control precautions for HRMO positive residents

In general, when giving physical care to residents, healthcare workers (HCWs) should always take standard precautions, such as adequate hand hygiene. These are meant to reduce the risk of transmission of pathogens from both known and unknown sources. The standard precautions are the minimal precautions a HCW must take in the care of all residents [9–11].

The additional infection control precautions are described in Table 5a and b. In order to be clear and undisputable, all precautions are listed, including the standard precautions such as hand hygiene.

**Table 5a:** Standard and additional infection control precautions by HRMO positive residents, per HRMO in a non-outbreak setting

HRMO or indication	Hand hygiene^b^	Personal Protective Equipment			Single room/apartment	Sanitation		Use of shared facilities such as living room
		Gloves^c^	Apron/Single use isolation gown with long sleeves^c^	Mask		Toilet/commode chair	Bathroom	
Enterobacteriaceae (ESBL included, not CPE^a^)	Yes	Yes	Apron	No^d^	no	No sharing with other residents	Sharing possible^e^	Yes^f^
CPE^a^	Yes	Yes	Isolation gown	No^d^	Yes^g^	No sharing with other residents	No sharing with other residents	Depending on individual situation^h^
*Acinetobacter species*	Yes	Yes	Isolation gown	No^d^	Yes^g^	No sharing with other residents	No sharing with other residents	Depending on individual situation^h^
*Pseudomonas aeruginosa*	Yes	Yes	Apron	No^d^	no	No sharing with other residents	Sharing possible^i^	Yes^f^
*Stenotrophomonas maltophilia*								
*Streptococcus pneumoniae (PRP)*	Yes	Yes	Apron	FFP1^j^	Yes^g, k^	Sharing possible	Sharing possible^e^	Yes, under conditions^f, l^
*Enterococcus faecium* (VRE)	Yes	Yes	Apron	No^d^	Yes, by preference^g^	No sharing with other residents	No sharing with other residents	Yes^f^
Recent admission foreign HCF	Yes	Yes	Isolation gown	None^d, m^	Yes^g^	No sharing with other residents	No sharing with other residents	Depending on individual situation ^h, l^

^a^CPE: Carbapenem resistant Enterobacteriaceae

^b^WHO 5 moments of hand hygiene (part of standard precautions)

^c^Wear only when giving physical care to residents and/or contact with material of residents. Do not wear during pure social activities

^d^Use a surgical mask whenever there is a risk of splashing of body fluids (part of standard precautions)

^e^When resident shares a bathroom: HRMO positive resident is the last one to use the bathroom, immediate cleaning procedure after use

^f^Give resident instruction on how to perform hand hygiene. If residents have wounds or indwelling catheters, cover these with an appropriate (wound)dressing

^g^If HRMO positive resident shares his room or apartment with another resident (e.g. couples), the other resident is also to be considered as an HRMO positive resident

^h^Consult the medical microbiologist and/or infection control practitioner to determine if visiting shared facilities (e.g. living room) is appropriate (taking into account the risk of transmission to other residents). If using shared facilities is permitted, see footnote f

^i^If resident shares bathroom facilities, ensure that after use immediate cleaning and disinfection takes place (important issue because of long survival duration of these microorganisms)

^j^FFP: Filtering facepiece particle; Put FFP1 mask in place before entering resident’s room (only in the acute phase of a respiratory infection which means, in the first 48 hours after appropriate antibiotic treatment has started)

^k^Single room/apartment is only necessary in the acute phase of a respiratory infection, which means in the first 48 hours after appropriate antibiotic treatment has started

^l^Use of shared facilities not allowed in the acute phase of a respiratory infection, in the first 48 hours after appropriate antibiotic treatment has started

^m^Special note: When a resident is suspected of MRSA carriage (e.g. after recent admission in foreign HCF, use surgical mask as part of the precautions for prevention of MRSA)

**Table 5b:** Standard and additional infection control precautions by HRMO positive residents, per HRMO in a non-outbreak setting

HRMO or indication	Materials, instruments, devices used for care of HRMO positive resident	Cleaning and disinfection of room/bathroom^n^			Disposal of materials
		Cleaning	Disinfection	Terminal cleaning^o^/ terminal disinfection^p^	Waste or Laundry
Enterobacteriaceae (ESBL included, not CPE^a^	No sharing with other residents^q, r^	Daily	Yes, if used for other residents as well^s^	Terminal cleaning procedure	Disposal in closed, intact bag
CPE^a^	No sharing with other residents^q, r^	Daily^t^	Yes, if used for other residents as wel^s^	Terminal disinfection procedure	Disposal in closed, intact bag
*Acinetobacter species*	No sharing with other residents^q, r^	Daily^t^	Yes, if used for other residents as well^s^	Terminal disinfection procedure	Disposal in closed, intact bag
*Pseudomonas aeruginosa*	No sharing with other residents^q, r^	Daily	Yes, if used for other residents as well^s^	Terminal disinfection procedure, only sanitation room(s)	Disposal in closed, intact bag
*Stenotrophomonas maltophilia*					
*Streptococcus pneumonia* (PRP)	No sharing with other residents^q, r^	Daily	Yes, if used for other residents as well^s^	Terminal cleaning procedure	Disposal in closed, intact bag
*Enterococcus faecium* (VRE)	No sharing with other residents^q, r^	Daily	Yes, if used for other residents as well^s^	Terminal disinfection procedure	Disposal in closed, intact bag
Recent admission foreign HCF	No sharing with other residents^q, r^	Daily^t^	Yes, if used for other residents as well^s^	Not applicable	Disposal in closed, intact bag

^n^For instructions for cleaning and disinfection of (bath) room: follow national guidelines

^o^Terminal cleaning (or end-cleaning): cleaning of room (all surfaces, touch surfaces, floor, splashing zones of walls) including bathroom and all re-usable materials present in this room. Re-usable materials which cannot be cleaned and non-reusable materials will be discarded of. Terminal cleaning takes place if additional precautions are discontinued or when resident is discharged, transferred or deceased

^p^Terminal disinfection (or end-disinfection): after terminal cleaning^o^, terminal disinfection of the room is performed (all surfaces, touch surfaces, floor, splashing zone of wall) including bathroom facilities and other re-usable materials present in the room (e.g. curtains, remote control, etc.). A terminal disinfection takes place after the additional precautions of the HRMO positive resident are discontinued, or the resident is discharged, transferred or deceased. Re-usable materials which cannot be disinfected, should be discarded of

^q^A small supply of necessary materials for the immediate care of the resident is allowed in the room/apartment. These materials must not be used for other residents and must be discarded after discharge or discontinuation of additional precautions

^r^If re-usable materials are used, they should be disinfected immediately when taken outside the resident’s room/apartment

^s^E.g. resident lifting devices, stethoscope

^t^Use cleaning material only for the room and devices of HRMO positive resident. Discard non-reusable cleaning material immediately after use. If the cleaning material is re-usable, remove materials after use in appropriate closed bag

### Documentation and communication of HRMO carriage

The documentation of the HRMO carriage is of utmost importance. Without knowing this, precautions to prevent transmission in the LTCF and other healthcare facilities (HCFs) cannot be taken. Therefore, all HCW involved, including those who are involved outside the LTCF (e.g. treating physicians in a hospital, primary care physicians), should be informed of the HRMO status of the resident. In addition, the HRMO status should be documented in the (E) Health records for (para) medical and nursing staff.

Before transferring a HRMO positive resident to another ward/small-scale living group within the facility, or another facility, or before visiting e.g. an outpatient department, all those providing care should be informed about the HRMO status.

When a HRMO positive resident is re-admitted to a LTCF and there have not been 2 sets of negative HRMO cultures according to the rules mentioned in the section “Discontinuation of additional infection control precautions” below, additional precautions should be taken.

The HRMO carriers themselves/or the first contact person and their caregivers should be notified about the HRMO status in order to receive needed information with regard to the consequences as well as being able to apply adequate infection control measures. It is necessary that the physician-in-charge and other HCWs of the LTCF have the opportunity to consult a medical microbiologist and/or an infection control practitioner for advice regarding the prevention of transmission, diagnostics and treatment for HRMO positive residents.

### Discontinuation of additional infection control precautions

Based on experience from earlier outbreaks and expert opinion, additional infection control precautions can be discontinued in the following cases:

- Resident, suspected for HRMO carriage:
  - ○ If the HRMO screening cultures (Table 1) are negative, additional precautions can be discontinued. The resident should be without antibiotic treatment for at least 48 h before cultures are taken.
- Resident, HRMO positive
  - ○ If a resident is carrier of Enterobacteriaceae, (Extended-spectrum beta-lactamase (ESBL) included, Carbapenemase-producing Enterobacteriaceae (CPE) excluded), *Acinetobacter* species, *Pseudomonas aeruginosa*, *Stenotrophomonas maltophilia* and *Streptococcus pneumonia* (PRP), then additional precautions can be discontinued if at least 2 sets of HRMO screening cultures (taken at least 24 h apart) are negative.
  - ○ If a resident is carrier of CPE or vancomycin-resistant *Enterococcus faecium* (VRE), additional precautions can be discontinued if at least 2 sets of cultures are negative, at least 1 year apart.

### Contact tracing of HRMO

Contact tracing is recommended in case of unexpected HRMO positive residents. When a contact of the HRMO positive index appears to be HRMO positive too, it could be due to a single transmission event or it can be the result of broad transmission within a facility. In order to detect and prevent further transmission, contact tracing is recommended for all HRMO, possibly with an exception for ESBL positive Enterobacteriaceae or for Enterobacteriaceae resistant for Quinolones and Aminoglycosides. These two HRMOs are commonly found in the Dutch population with a prevalence of up to 8–10% in patients seeing a general practitioner [12].

The scale of the contact tracing is determined by the elderly care physician in collaboration with the medical microbiologist and/or infection control practitioner. In most cases, all residents who have been in contact with the HRMO positive resident will be cultured for HRMO carriage (see Table 4). Additional precautions can be postponed until the culture results from the first investigation are known. Directly changing precautions have much impact for the residents and HCWs and according to expert opinion is not advisable until transmission actually has been proven. If residents are transferred to another ward or HCF, it is advised to take additional precautions while waiting for culture results. Residents who are already discharged to their home-setting, will initially not be cultured unless in the first investigation HRMO positive residents are detected.

Contact tracing is also recommended if HRMO carriage is confirmed with a HRMO suspected resident and it is known that the additional precautions have not adequately been performed in the time between culturing and results. In that time HRMO transmission could have taken place.

Contact tracing among HCWs is not indicated. HCW, if at all, are only transient carriers of HRMO. In addition - and in contrast to MRSA - possibilities for decolonization treatment of HRMOs is limited and not routinely used.

### Environmental control/investigation

Initially, culturing the environment to detect a source of the HRMO is not indicated. If, however, during an outbreak with HRMO transmission persists, environmental culturing may be considered to determine a source of the outbreak.

### Surveillance of HRMO

Evaluation of the local and regional epidemiology of HRMO provides knowledge in the, sometimes rapidly changing, evolution in this area.

Performing surveillance on a local and regional level, by exchanging on a regular basis HRMO data from routine diagnostics, can be very helpful to determine if there is an indication of an increasing level of HRMO. At present, gathering and combining data to receive insight into the regional epidemiology is a task for the Dutch regional networks, initiated by the ministry of health, to combat AMR. To have unbiased surveillance data (at least once a year) point-prevalence studies among the residents of the LTCF should be performed by trained professionals to determine local levels and possible transmission of HRMO in the facility.

### Outbreak

A situation is considered to be an outbreak when 2 or more residents have the same HRMO and the presence of an epidemiological link between them.

During an outbreak, it is important to maintain and highlight the standard precautions and additional precautions specific for that kind of HRMO (as described in Table 5b).

It is strongly advisable to install an Outbreak Management Team. This multidisciplinary team consists an elderly care physician, medical microbiologist, infection control practitioner, staff members of the wards involved (both nursing and medical), member of the management team and professional of the Local Health Authority.

This team which will take care of the arising issues such as decisions on additional infection control precautions, adjustment of antibiotic therapy, communication within and outside of the LTCF where the outbreak takes place and alert national authorities of this specific outbreak.

When despite tightening up infection prevention precautions, further transmission takes place, confirmation of clonal relationship between the strains or plasmids by molecular typing needs to be done [13].

## Discussion

There are limitations to this guideline for HMRO carriage in long term care settings.

First, the absolute risk of transmission of HRMO within the Dutch LTCFs (as defined in the guideline) is not known. However, there is a growing understanding of the potential for transmission of HRMO in the LTCF. In 2016, den Dool et al. used mathematical modelling to estimate the contribution of nursing homes in the dispersal of pathogens over the healthcare network in the Netherlands. They concluded that nursing homes have the potential to drive and sustain epidemics across this network and that infection control efforts and surveillance systems should also be targeted at those LTCFs [14]. Recent research in Dutch LTCFs showed that, although in absolute numbers the percentage of HRMO is low (4.2% *Escherichia coli* ESBL carriage among residents), the large variation of HRMO presence between facilities (1–33%) warrants cautious surveillance [15].

Secondly, it is not known how long a resident remains colonised with HRMO. Research shows that carriage can persist over years, depending on the micro-organism [15]. The guidance for the decision to discontinue infection control precautions is therefore based on expert opinion. Although research indicates that there might be predisposing factors for prolonged carriage, more research is needed to determine when to discontinue precautions and consider HRMO carriage as ended in long-term care. Despite the lack of studies that show the effect of monitoring of the HRMO carriage of a resident and its consequences in LTCFs, it is logical to assume that these measures are effective to prevent the transmission of HRMO.

Last but not least, it is not known whether the proposed actions in the LTCF on prevention of transmission of HRMO, are equally effective and achievable for the various groups of residents in such facilities, such as e.g. psychogeriatric residents. However, given the rising evidence for spreading of HRMO within the LTCF settings, this is a first step in developing guidelines for prevention of transmission of HRMO. Over the course of time, with leaders in both infection control and LTCFs, further guidance should be provided, while the absolute risk of transmission and harm as opposed to the adverse events related to additional precautions, such as reduced psychological wellbeing, resident safety and satisfaction in residential care [7].

## Data Availability

Not applicable.

## Abbreviations

AMR: Antimicrobial resistance
CPE: Carbapenemase-producing Enterobacteriaceae
ESBL: Extended-spectrum beta-lactamase
HCFs: Healthcare facilities
HCWs: Healthcare workers
HRMO: Highly-resistant micro-organisms
LTCFs: Long-term care facilities
MRSA: Methicillin-resistant *Staphylococcus aureus*
PRP: *Streptococcus pneumonia*
VRE: *Enterococcus faecium*
WHO: World Health Organization
WIP: Dutch Working Party for Infection Control
